# Role of vitamin D receptor and calcium sensing receptor in parathyroid cancer

**DOI:** 10.1016/j.bjorl.2026.101848

**Published:** 2026-06-18

**Authors:** Dongxue Zhang, Teng Zhao, Dehui Bi, Jian Huang, Tao Jiang, Pengxiang Zhao, Bojun Wei

**Affiliations:** a Capital Medical University, Beijing Shijitan Hospital, Department of Endocrinology, Beijing, China; b Capital Medical University, Beijing Chaoyang Hospital, Department of Thyroid and Neck Surgery, Beijing, China; c Beijing University of Technology, Faculty of Environment and Life, Beijing, China

**Keywords:** VDR, CaSR, lncRNA, Hyperparathyroidism, Parathyroid cancer

## Abstract

**Objectives:**

Role of Vitamin D Receptor (VDR), Calcium Sensing Receptor (CaSR) and their associated long non-coding RNAs (lncRNAs) in Parathyroid Cancer (PC) development and PC diagnosis need to be explored. LncRNA SLC2A1-DT and SLC22A5-AS1 were dysregulated in previous microarray study. LncRNA SLC2A1-DT may target on VDR in prediction. Meanwhile, lncRNA SLC22A5-AS1was associated with CaSR.

**Methods:**

12 patients with PC and 43 patients with Parathyroid Adenoma (PA) were enrolled in this study. Fresh tissue samples were collected. Quantitative Real-Time Polymerase Chain Reaction (RT-qPCR) were used to assess the mRNA expression level of VDR and CaSR. In addition, protein level of VDR and CaSR were evaluated by Immunofluorescence (IF) detecting. Furthermore, expression level of lncRNA SLC2A1-DT, which may target on VDR in prediction, was validated by RT-qPCR in this study. Meanwhile, expression level of lncRNA SLC22A5-AS1, which was associated with CaSR in previous microarray study, was also detected by RT-qPCR.

**Results:**

Down-regulated mRNA expression levels of VDR (p = 0.003) and CaSR (p = 0.000) were detected in patients with PC. Consistent with the mRNA profile, protein expression levels for these two genes were also decreased in PC group in IF detection. In addition, association between VDR and CaSR were also confirmed in patients with hyperparathyroidism (p = 0.000). However, there was no significant difference in Area Under Curve (AUC) among VDR, CaSR and PTH in parathyroid cancer diagnosis. Meanwhile, there was no difference in lncRNA SLC2A1-DT and SLC22A5-AS1 expression levels in RT-qPCR validation. However, expression levels of lncRNA SLC2A1-DT (p = 0.004) and SLC22A5-AS1 (p = 0.002) were increased in PC patients with normal serum phosphorus. In patients with normal serum phosphorus, the AUC of SLC2A1-DT was 0.896 (p < 0.001). Similarly, the AUC of SLC22A5-AS1 was 0.920 (p < 0.001).

**Conclusion:**

Decreased VDR and CaSR may play a role in parathyroid cancer. VDR and CaSR may have interaction in hyperparathyroidism. LncRNA SLC2A1-DT and SLC22A5-AS1 were dysregulated in PC patients with normal phosphorate. These lncRNAs could serve as potential biomarkers for PC patients with normal phosphorus.

**Level of evidence:**

This diagnostic or monitoring test accurate? (Diagnosis).

**Level 4:**

Case-control studies.

## Highlights

• VDR and CaSR were downregulated in parathyroid cancer.
• VDR and CaSR participate in parathyroid cancer development.
• VDR and CaSR may have interaction in parathyroid cancer.
• LncRNAs associated with VDR may act as diagnostic bimomarkers.

## Introduction

Primary Hyperparathyroidism (PHPT) is a common disease in endocrine system, which is characterized by an excessive production of Parathyroid Hormone (PTH) and hypercalcemia. PHPT was primarily found in patients diagnosed with Parathyroid Adenoma (PA), while Parathyroid Cancer (PC) was extremely rare. It has been found that PC was resistant to radiation and chemotherapy.^1^ Hence, the curative treatment of parathyroid malignancy could be obtained only when complete excision was achieved during the first surgery.^2^ To find more effective diagnosis and treatment for PC, it is important to explore the mechanism for PC development. However, molecular mechanism for PC is still not well elucidated.

Impaired Vitamin D Receptor (VDR) and Calcium Sensing Receptor (CaSR) play a vital role in parathyroid tumors.^3^ VDR and associated complex can bind with the vitamin response element to regulate PTH synthesis.^4^ Meanwhile, PTH also can be regulated by the CaSR.^4^ In addition, A decrease expression of VDR and CaSR was found in PA,^3^^,^^5^ which also participate in parathyroid cell proliferation.^6^ Further, the protective effect of VDR on human cancers has been widely reported.^7^ Meanwhile, reduced CaSR has been reported to be associated with an increased risk for recurrence a distant metastasis in patients with PC.^8^ However, difference of VDR between PC and PA is still unknown.

Interestingly, downregulation of VDR and CaSR were found in PC in our previous lncRNA-mRNA micro-array study.^9^ However, how the expression of VDR and CaSR is regulated in parathyroid tumors remains to be explored. Genetic mutations in these genes appear to be uncommon in sporadic tumors.^10^ Epigenetic modifications, such as promoter methylation, have been investigated but could not fully explain the reduced expression of VDR and CaSR in PAs.^11^ This suggests the involvement of other regulatory mechanisms. Long non-coding RNAs (lncRNAs), as important epigenetic regulators, represent a potential avenue for investigation. Our previous lncRNA-mRNA microarray study identified downregulation of both VDR and CaSR in PC.^9^ Re-analysis of this microarray data (unpublished) revealed that two down-regulated lncRNAs, SLC2A1-DT and SLC22A5-AS1, were computationally predicted to be associated with VDR and CaSR, respectively. This preliminary bioinformatic association prompted us to investigate their expression in a clinical cohort. Therefore, this study aimed to preliminarily examine the expression patterns of VDR, CaSR, and these candidate lncRNAs in PC versus PA, and to explore their potential correlations.

In the current study, VDR and CaSR were compared among patients with PC and PA. Further, this paper also explores the preliminary diagnostic value of VDR and CaSR in PC. In addition, validations of lncRNA SLC2A1-DT and SLC22A5-AS1 associated with VDR or CaSR were also performed in this cohort.

## Methods

### Patients and samples

Tissue samples were obtained from patients with sporadic primary hyperparathyroidism during operations. 12 patients with parathyroid carcinoma and 43 subjects with PA were enrolled. Two pathologists conducted the histopathological diagnosis separately. The diagnosis of PC was established when postoperative histopathological examination confirmed the presence of invasive features, including angioinvasion, perineural invasion, local invasion (e.g., adjacent soft tissues, musculoskeletal structures, thyroid gland, and esophagus), lymph node metastasis, or distant metastasis. Fresh samples were immediately put in liquid nitrogen and frozen at −80 °C. At the same time, basic clinical information was also collected. This subject setting consisted of 20 males and 35 females. The mean age of these patients was 57.3 ± 14.1. The current study was approved by the Institutional Review Board (IRB) of Ethics Committees. Written informed consent was collected from participants.

Given the rarity of parathyroid carcinoma, collecting large clinical cohorts is challenging. Our sample size (12 PC, 43 PA) is comparable to or larger than several previously published molecular studies focusing on PC (e.g., Witteveen^8^ et al., Mod Pathol 2011, n = 10 PC). This preliminary investigation aims to generate hypotheses for future validation in larger, multi-center cohorts.

### Total RNA purification and reverse transcription

According to the manufacturer's instructions, total RNA was extracted from the frozen tissue sample with 1 mL Trizol Reagent (Invitrogen, Thermo Fisher Scientifific, Waltham, MA, USA) as we described before.^9^^,^^12^ A NanoDrop® ND-1000 spectrophotometer (Thermo Fisher Scientific, Waltham, MA, USA) was used in RNA quality assessment. OD A260/A280 ratio for total RNAs was close to 2.0. In addition, an Agilent 2100 Bioanalyzer (Agilent Technologies, Santa Clara, CA, USA) was used in RNA integrity evaluation. RIN were greater than 7.0 and 28 s/18 s were greater than 0.7. A High-Capacity cDNA Reverse Transcription Kit (Applied Biosystems) was used in reverse transcription of RNA following the manufacturer’s instruction.

### RT-qPCR

All mRNAs and lncRNAs were assessed by a Reverse-Transcription quantitative Polymerase Chain Reaction (RT-qPCR). Following the manufacturer's instructions, the 2 × PCR master mix (Arraystar) was used on a ViiA 7 real-time PCR system (Applied Biosystems) to complete PCRs. To validated the primers, melting curves was conducted (temperature range: 60–95 °C, heating rate: 0.1 °C/s) (absence of additional peaks). Glyceraldeyde-3-Phosphate Dehydrogenase (GAPDH) was selected as an internal control. At the beginning, PCR reactions were performed under a 95 °C denaturation lasting for 10 min. Forty cycles followed, which conducted at 95 °C for 10 s, 60 °C for 60 s, and 95 °C for 10 s. −ΔCT or 2^−ΔΔCT^ was calculated for the comparison in relative expression levels. For each target gene and internal control, each cDNA sample was detected in triplicate in the same RT-qPCR run. In addition, tissue samples from all enrolled subjects were included in the experiment.

### Immunofluorescence and Hematoxylin and Eosin (H&E) staining

Immunostaining was conducted on parathyroid tumor sections prepared from paraffin blocks. 2–3 μm slices were obtained from tissue section. The sections were blocked overnight at 4 °C, washed and incubated for 90 min at 37 °C with VDR Monoclonal antibody and CaSR antibody (proteintech, 67192−1-Ig; proteintech, 19125-1-AP). Washings with PBS and incubation in dark at 37 °C for 1 h with the appropriate fluorescent dye-labeled secondary antibodies (Alexa Fluor R488 Conjugate anti-rabbit IgG (H + L), 1:1,000, Cell Signaling, #4412; Alexa Fluor R594 Conjugate anti-rat IgG, 1:1000, Abcam, ab150160) were followed. NucBlue Live cell stain (R37605, Invitrogen) was used for cell nuclei staining as recommended by the manufacturer. The sections were analyzed under a Lica microscope.

Paraffin-embedded tissues sections were hydrated. Subsequently, the tissue sections were dipped in hematoxylin, agitated for 30 sec and rinsed in H_2_O for 1 min. The slides were further stained by immersion in 1% eosin Y solution for 10–30 sec with agitation. After this step, the sections were dehydrated. At the end of the staining processes, drops of mounting medium were added on the slides, covered with a coverslip and analyzed under a Lica microscope.

### Statistical analysis

Statistical analyses were performed using SPSS (Version 17.0, Chicago, IL, USA), GRAPHPAD PRISM (Version 8.0 La Jolla, CA, USA), or MedCalc Software (Version 15.8, Mariakerke, Belgium). Quantitative data were shown as mean ± SEM, while numbers or percentages were used in qualitative data presentation. Comparison of the means between PC and PA was conducted with Mann-Whitney *U* test or Student’s *t*-test according to data distribution. Diagnostic values of RNAs in PC were assessed with Receiver Operating Characteristic (ROCs) curves without adjustment variables. Maximizing the Youden index was used in the determination of the optimal Cutoff value. Areas under ROC Curves (AUCs), sensitivity, specificity and Cutoff value were calculated in ROC cure analysis. Comparison of AUCs of ROC curves were performed using *Z* test. The comparison was shown as Z value. Spearman’s rank correlation was used in relevance analysis. Statistical significance was defined as p < 0.05.

## Result

### Down-regulated VDR and CaSR in PC

**Fig. 1:**
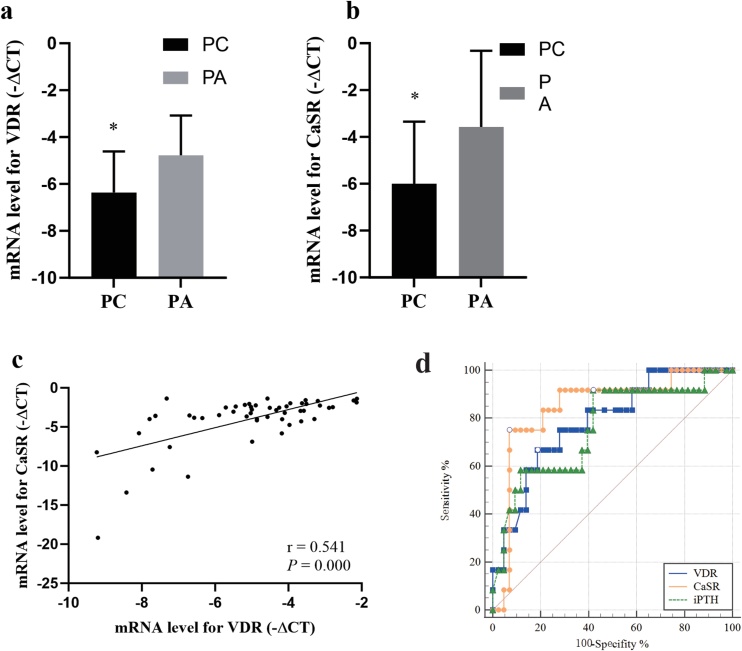
Quantitative reverse transcription Polymerase Chain Reaction (qRT-PCR) analyses of VDR and CaSR. mRNA levels for (a) VDR and (b) CaSR were lower in patients with Parathyroid Cancer (PC) than those with Parathyroid Adenoma (PA). (c) The mRNA profile for VDR was positively correlated with that for CaSR. (d) Receiver Operating Characteristic Curves (ROC) of VDR, CaSR, and iPTH in PC diagnosis. (d) There was no significant difference between the Areas Under Curve (AUC) of these indexes. * p < 0.05.

**Fig. 2:**
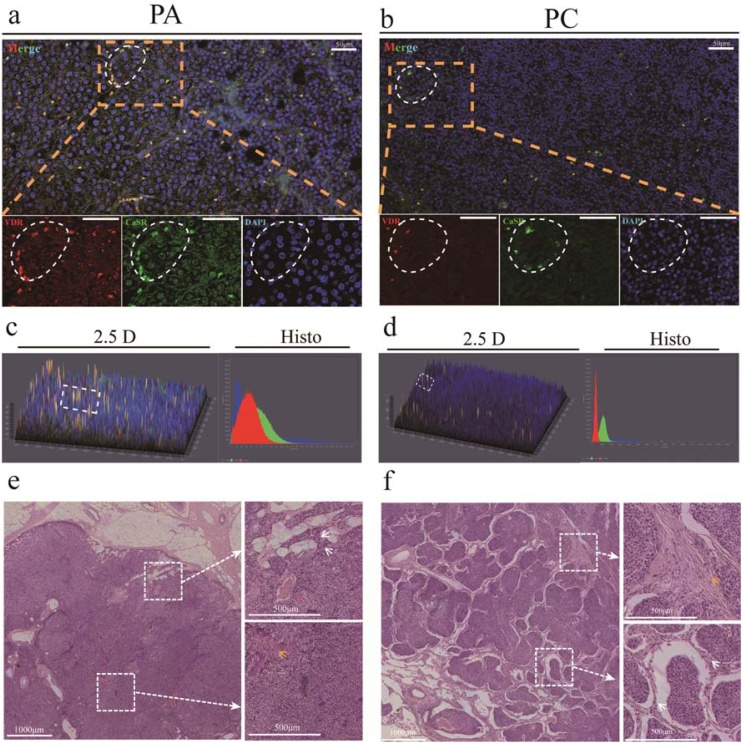
Immunofluorescence detection of vitamin D receptor (VDR) and Calcium-Sensing Receptor (CaSR). The VDR protein is represented by red fluorescence, and the CaSR protein by green fluorescence. The fluorescence signals for the VDR (a and c) and CaSR (b and d) were more intense in Parathyroid Adenoma (PA) tissues than that in Parathyroid Cancer (PC) tissues. Hematoxylin and eosin staining for PA (e) and PC (f).

To analyze the VDR and CaSR expression profile in parathyroid tumors, mRNAs of VDR and CaSR were detected from all samples. The mRNA level of VDR was significantly decreased in patients with PCs compared with those with PAs (Fig. 1a, p = 0.003). Similarly, the mRNA expression level of CaSR was also downregulated in PC (Fig. 1b, p = 0.000). Additionally, mRNA expression of VDR was positively correlated with that of CaSR (Fig. 1c, *rs* = 0.541, p = 0.000). VDR and CaSR protein levels were determined by IF in 2 PAs and 3 PCs. The protein levels of VDR and CaSR in PC were lower than those in PA (Fig. 2).

### Role of VDR and CaSR profile in the risk prediction for PC

In univariate analysis, both VDR (Table 1, p = 0.004) and CaSR (Table 1, p = 0.032) were associated with PC. Furthermore, lower levels of VDR (Table 1, p = 0.014) and CaSR (Table 1, p = 0.031) remained independent risk factors when adjusted by sex and age. However, lncRNA SLC2A1-DT was not an independent risk factor for PC after adjusted for sex, age, iPTH, and serum calcium.

**Table 1:** Associations between parathyroid cancer and different genes via logistic analysis.

Parathyroid cancer diagnosis	Model	OR	95% CI	p-value
VDR	Model 1	0.563	0.382‒0.830	0.004^a^
	Model 2	0.596	0.395‒0.899	0.014^a^
	Model 3	0.650	0.417‒1.014	0.057
CaSR	Model 1	1.253	1.020‒1.538	0.032^a^
	Model 2	0.796	0.647‒0.979	0.031^a^
	Model 3	0.831	0.674‒1.026	0.085

Model 1: Monovariate analysis.

Model 2: Adjusted with gender and age.

Model 3: Adjusted with gender, age, iPTH and Ca.

a p < 0.05 for logistic regression models.

The AUC (Fig. 1d) of VDR reached 0.786 (95% CI 0.654‒0.885, p = 0.0001) with a cutoff value of -6.35, sensitivity of 66.7%, and a specificity of 81.4%. For CaSR, the AUC was 0.847 (95% CI 0.724‒0.930, p < 0.0001), corresponding to a cutoff value of -4.75, a sensitivity of 75.0%, and a specificity of 93.0%. Meanwhile, The AUC of iPTH was 0.762 (95% CI 0.628‒0.866, p = 0.0019) with a cutoff value of 209, a sensitivity of 91.7%, and a specificity of 58.1%. Notably, there was no significant difference in AUCs among VDR, CaSR and PTH. The comparison between VDR and CaSR in AUC showed no significant difference (*Z* = 0.685, p = 0.493). Additionally, neither the AUC of VDR (*Z* = 0.302, p = 0.762) nor that of CaSR (*Z* = 0.742, p = 0.458) differed significantly from the AUC of PTH. These results suggest that VDR and CaSR expression, alongside PTH, may hold preliminary diagnostic value, though their clinical utility requires further assessment in larger studies.

### Profiles for LncRNAs and their association with VDR/CaSR

**Fig. 3:**
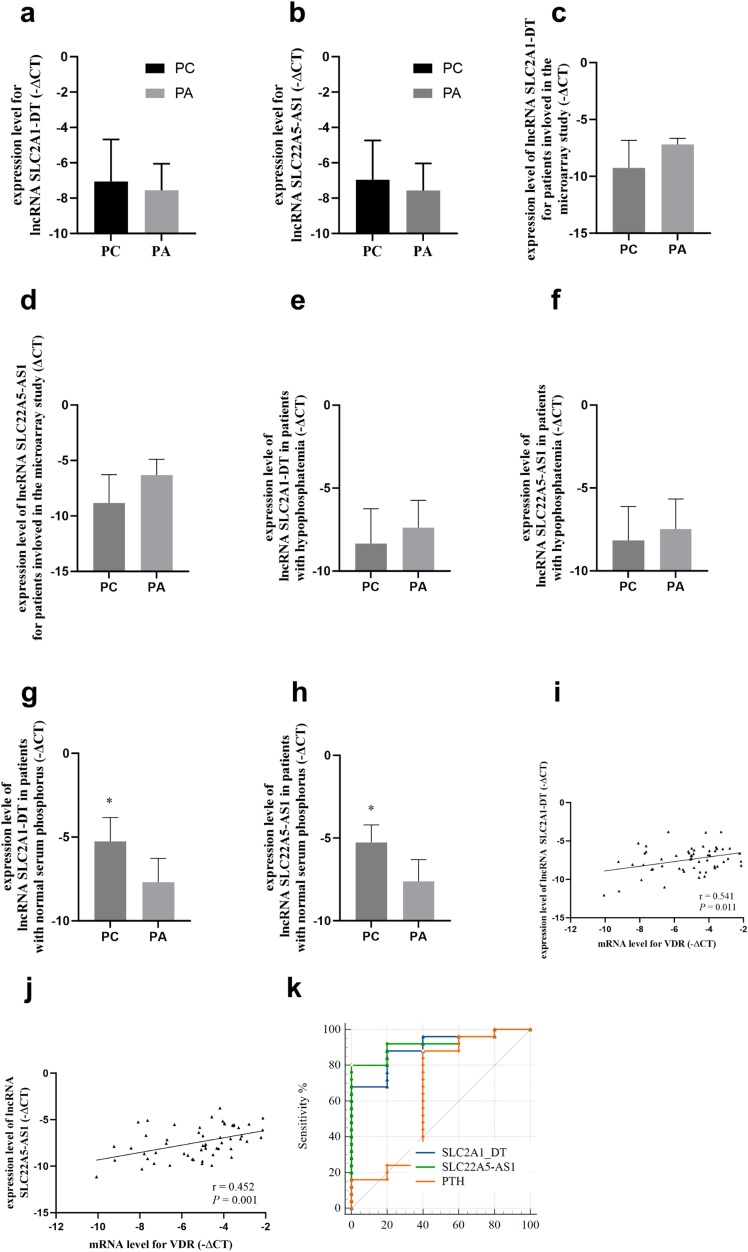
Quantitative reverse transcription polymerase chain reaction validation (qRT-PCR) of potential lncRNAs associated with VDR or CaSR. (a) The expression levels of the long non-coding RNA (lncRNA) SLC2A1-DT did not differ between patients with PC and PA. (b) The expression level of the lncRNA SLC22A5-AS1 did not differ between patients with PC and PA. (c and d) The expression levels of SLC2A1-DT (c) and SLC22A5-AS1 (d) tended to be downregulated in 4 PC patients compared with 3 PA patients included in the previous microarray study. (e and f) In patients with hypophosphatemia, the expression levels of SLC2A1-DT (e) and SLC22A5-AS1 (f) tended to be downregulated in PC compared with those in PA. (g and h) In patients with normal serum phosphorus, the expression levels of SLC2A1-DT (g) and SLC22A5-AS1 (h) were upregulated in PC compared with those in PA significantly. (i and j) The expression levels of SLC2A1-DT and SLC22A5-AS1 were correlated with that of VDR significantly. (k) ROC curves of SLC2A1-DT, SLC22A5-AS1, and PTH in patients with normal serum phosphorus. * p < 0.05.

In the RT-qPCR validation involving all patients, no significant difference was observed in the expression of SLC2A1-DT or SLC22A5-AS1 between PAs and PCs (Fig. 3a‒b). Given the small sample size and the exploratory nature of this study, we performed post-hoc analyses based on serum phosphate levels, a clinically relevant parameter in PHPT. In the subgroup of patients with normal serum phosphate (n = 20 PA, 5 PC), the expression levels of both SLC2A1-DT (Fig. 3g, p = 0.004) and SLC22A5-AS1 (Fig. 3h, p = 0.002) were significantly higher in PC compared to PA. However, this finding should be interpreted with caution due to the very small number of PC cases in this subgroup and its post-hoc nature. No significant differences were found in the hypophosphatemia subgroup (Fig. 3e‒f), likely due to limited power.

At the correlation level, a preliminary association was observed between SLC2A1-DT expression and VDR expression (Fig. 3i, r_s_ = −0.339, p = 0.011). SLC22A5-AS1 expression also showed a negative correlation with VDR (Fig. 3j, r_s_ = −0.452, p < 0.001). No significant correlation was found between SLC22A5-AS1 and CaSR expression in this cohort.

Exploratory diagnostic analysis within the normal serum phosphate subgroup showed high AUC values for both lncRNAs (SLC2A1-DT: 0.896; SLC22A5-AS1: 0.920). While these AUCs were numerically higher than that of PTH (0.648), the differences were not statistically significant (p > 0.05 for both comparisons), highlighting the need for validation in an independent and larger sample set.

### Associations between genes and clinical characteristics

Higher iPTH concentration (Table 2, p = 0.027) and longer disease course (Table 2, p = 0.048) were detected in patients with lower VDR expression. Except longer disease course (p = 0.025), no other different index was found in patients with lower CaSR (Table 2).

**Table 2:** Clinical characteristics in patients with different VDR or CaSR levels.

Clinical characteristics	Lower VDR (n = 27)	Higher VDR (n = 2 8)	p-value	Lower CaSR (n = 27)	Higher CaSR (n = 28)	p-value^b^
Age (SD)(years)	55.3(3.2)	59.1 (2.1)	0.692	52.7 (2.5)	61.6 (2.6)	0.011^a^
Sex (% M)	44.4%	28.6%	0.269	44.4%	28.6%	0.269
Disease course (SD) (month)	74.1 (17.2)	51.9 (18.4)	0.048^a^	76.4 (17.5)	49.7 (18.1)	0.025^a^
Symptomatic hyperthyroidism (%)	74.1%	60.7%	0.391	74.1%	60.7%	0.391
iPTH (SD) (pg/mL)	626.0 (128.3)	240.1 (39.0)	0.027^a^	575.6 (128.1)	288.7 (52.8)	0.162
Ca^2+^ (SD) (mmoL/L)	2.93 (0.11)	2.75 (0.06)	0.444	2.90 (0.11)	2.78 (0.05)	0.899
P (SD) (mmoL/L)	0.84 (0.05)	0.80 (0.05)	0.807	0.84 (0.05)	0.80 (0.05)	0.555
ALP (SD) (U/L)	190.0 (51.3)	107.9 (8.7)	0.378	192.11(51.0)	105.7 (9.7)	0.088
Maximum diameter (SD) (cm)	2.00 (0.25)	1.98 (0.23)	0.866	1.76 (0.20)	2.22 (0.27)	0.266

iPTH, Intact Parathyroid Hormone; Ca, Serum total Calcium; P, Serum Phosphorus; ALP, Alkaline Phosphatase.

a p < 0.05 for Mann–Whitney *U* test.

b p < 0.05 for χ2 test.

## Discussion

Pathogenesis of sporadic PC remains unclear. In addition, differentiating parathyroid carcinoma from benign tumors was still a challenge in clinical practice. According to the histologic criteria, lots of patients cannot be diagnosed after first operation until perineural or local invasion, vascular, lymphatic or distant metastasis occurs.^13^ Recently, loss of parafibromin or mutation in CCDC73 may participate in PC diagnosis, but it has not shown a desirable efficiency.^14^ Moreover, parafibromin only can be detected via immunohistochemical detecting after operation. In addition, PC was resistant to radiation and chemotherapy.^1^ Hence, investigating PC development mechanisms is important for PC diagnosis and treatment.

In this study, decreased VDR level was detected in PC compared with PA. Compared with normal parathyroid gland, down regulated VDR was found in PA and secondary hyperparathyroidism.^3^^,^^15^ On one hand, VDR binding with calcitriol has been found to inhibit PTH secretion.^16^^,^^17^ Consistent with these findings, lower VDR level was associated with excessive PTH secretion in our study, indicating a weakened ability of VDR to inhibit PTH secretion. In the other hand, reduced VDR has found to be associated cell proliferation in PA.^3^ It has been shown that VDR was negatively associated with ki-67 level in PA.^3^ In this work, we found that mRNA level of VDR was also decreased in PC compared with that in adenoma. In agreement with our study, VDR expression in colorectal cancer tissues was lower than that in normal tissues. In patients with colorectal cancer, a moderate diagnostic performance was generated from VDR (AUC = 0.88). In addition, lower VDR expression was also recognized as a significant prognostic predictor in colorectal cancer.^18^ A decreased expression of VDR has been shown in adrenocortical carcinoma tissues.^19^ Hence, lower VDR may participate in PC development.

In the current study, CaSR was also downregulated in PC compared with benign parathyroid tumors. Loss of CaSR expression was more frequent in PC than that in PA or normal parathyroid tissue,^20^ and lower CaSR level was a negative prognostic factor for PC.^8^ Reduced CaSR expression promoted cell proliferation in cancers,^21^ and CaSR functioned as a tumor suppressor in malignancies of the nervous system.^22^ Diminished CaSR weakened its ability to inhibit PTH secretion and may also contribute to parathyroid gland hyperplasia.^8^ Thus, CaSR may be involved in the pathological progression of PC.

In this work, mRNA level of VDR was correlated with that of CaSR in patients with hyperparathyroidism. This is consistent with a previous finding that VDR protein level was associated with CaSR protein level in highly proliferative PA,^3^ suggesting potential interactions between VDR and CaSR in parathyroid tumors. In normal rat parathyroid cells, extracellular calcium binding to CaSR increased VDR expression, which may be mediated via the ERK1/2-MAPK signaling pathway.^23^ Additionally, VDR could upregulate CaSR expression in the parathyroid by binding to vitamin D response elements in the CaSR promoter (P1 and P2 regions).^24^ Furthermore, an integrated transcriptome and whole-exome sequencing study identified VDR and CASR as key genes distinguishing PA from normal parathyroid tissues.^10^ Taken together, reduced VDR and CaSR expression may interact and play critical roles in PC initiation.

The mechanism underlying VDR and CaSR downregulation in PC require further elucidation. Mutations in CaSR, but not VDR, were found in parathyroid neoplasm^10^ in previous study. Recently, only one study revealed the VDR mutation in Greek population with hyperparathyroidism.^25^ Reduced VDR expression level in adrenocortical carcinoma maybe resulted from the methylation of CpG sites in the promotor of VDR. Hence, epigenetic modifications also need to be detected to explain why VDR reduced in patients with hyperparathyroidism. In parathyroid adenoma, no significant methylation was found in the promoter regions of VDR and CASR genes.^11^ However, in a recent study, increased histone modification and hypermethylation of the CaSR promoter were found in PA.^5^ In this study, lncRNAs associated with VDR as well as CaSR were researched, which may participate in VDR regulation.

The expression profiles of both lncRNA SLC2A1-DT and SLC22A5-AS1 in PC did not differ from those in PA. Consistent with the previous microarray study,^9^ RT-qPCR validation performed on the same patients involved in the microarray analysis showed that the expression levels of these two lncRNAs tended to be lower in PC than in PA. Except for serum phosphate levels, no differences in clinical characteristics were observed between the PC patients included in the microarray study and those in the RT-qPCR validation cohort.^26^ Notably, in patients with hypophosphatemia, the lncRNAs were dysregulated in a manner consistent with the microarray results. But the difference was not statistically significant, possibly due to the small sample size. In contrast, in patients with normal serum phosphate, the expression of these lncRNAs was significantly elevated in PC.

In patients with hyperparathyroidism, hypophosphatemia has been associated with a higher risk of renal stones and osteoporosis, and may indicate a poor prognosis.^27^ Cancer cells have a high demand for phosphate to meet the requirements of rapid growth. Indeed, serum phosphate concentrations in cancer patients have been found to be higher than those in healthy persons.^28^ Additionally, phosphate was involved in the regulation of lncRNA expression.^29^ Collectively, these findings suggest that phosphate may contribute to the differing lncRNA expression profiles observed between the microarray study and RT-qPCR validation.

LncRNA SLC2A1-DT, also known as SLC2A1 Antisense RNA1 (SLC2A1-AS1), is a lncRNA located on chromosome 1p34.2. In this work, we found that SLC2A1-DT expression was increased in parathyroid malignant lesions PA in patients with normal serum phosphate. In lung adenocarcinoma patients, SLC2A1-DT expression was found to be higher and associated with poor long-term survival. It acted as an oncogene to drive cell proliferation in lung and pancreatic cancer cell lines.^30^ On the other hand, SLC2A1-DT is considered as a tumor suppressor gene in hepatocellular carcinoma progression by inhibiting Glucose Transporter-1 (GLUT1) expression.^31^ This indicates that SLC2A1-DT may play different roles in specific cancer types.^31^ In the current work, SLC2A1-AS1 was dysregulated in PC patients with normal serum phosphate. VDR was predicted as a trans-target gene of lncRNA SLC2A1-DT, and SLC2A1-DT expression was correlated with VDR expression. In addition, the Area Under the Curve (AUC) of SLC2A1-AS1 for PC diagnosis was up to 0.896. Hence, SLC2A1-AS1 may contribute to the diagnosis of parathyroid cancer in patients with normal serum phosphate.

LncRNA SLC22A5-AS1, also known as MIR3936-host gene (MIR3936-HG) or LOC553103, showed increased expression in PC patients with normal phosphate levels in this study. Serum SLC22A5-AS1 has been found to be a diagnostic and prognostic biomarker for 15 cancer types, such as lung cancer, hepatocellular carcinoma, thyroid cancer and et al.^32^ It also acted as an Extracellular Matrix (ECM) organizer, playing a critical role in cancer cell proliferation and adhesion.^32^ PC exhibited deregulated expression of ECM protein-encoding genes; the ECM gene network may serve as a hallmark of parathyroid tumor proliferation.^33^ Additionally, CaSR has been shown to be involved in ECM deposition in airway smooth muscle,^34^ while VDR was a key inhibitor of ECM production in pancreatic stellate cells.^34^^,^^35^ Our previous microarray study suggested an association between SLC22A5-AS1 and CaSR expression, but this association was not confirmed by RT-qPCR validation in the current study, possibly due to limited sample size. In contrast, RT-qPCR validation revealed a negative correlation between SLC22A5-AS1 and VDR. Thus, SLC22A5-AS1 may contribute to ECM production in PC by downregulating VDR, and could serve as an assistant diagnostic biomarker for PC.

### Study limitations and future perspectives

This study has several limitations that must be acknowledged. First, the sample size, particularly for the rare PC group (n = 12), is small. While this is comparable to other molecular studies in this field and reflects the disease's low incidence, it limits the statistical power and generalizability of our findings. The subgroup analyses based on serum phosphate levels, especially, involved very few PC cases and were exploratory (post-hoc) in nature. Therefore, the significant upregulation of lncRNAs in the normal phosphate subgroup and their high AUC values must be interpreted as preliminary observations generating a hypothesis for future testing, rather than definitive conclusions. Second, our study is primarily descriptive and correlative. We report associations between the downregulation of VDR/CaSR and PC, and between specific lncRNAs and VDR, but we cannot establish causality or define the underlying molecular mechanisms. The lack of functional validation (e.g., knockdown/overexpression experiments) for the candidate lncRNAs is a key gap. Third, the potential of SLC2A1-DT and SLC22A5-AS1 as diagnostic biomarkers is far from established. Cross-sectional, single-center design and small sample size preclude any assessment of their prognostic value. Future research requires validation in large, independent, multi-center cohorts with longitudinal follow-up.

To address these limitations, we plan to: (1) Expand our clinical cohort through multi-center collaboration; (2) Perform functional experiments in parathyroid cell models to investigate the mechanistic roles of VDR, CaSR, and the candidate lncRNAs; and (3) Explore the diagnostic utility of these markers in peripheral blood.

## Conclusion

In conclusion, this preliminary study demonstrates that decreased expression of VDR and CaSR is associated with parathyroid carcinoma and may contribute to its pathogenesis, likely through interacting pathways. Furthermore, we identified that the expression of lncRNAs SLC2A1-DT and SLC22A5-AS1 ‒ which show a preliminary correlation with VDR ‒ is altered in a subset of PC patients with normal serum phosphate. These findings highlight VDR and CaSR as potential players in PC biology and nominate SLC2A1-DT and SLC22A5-AS1 as candidate biomarkers for future validation. The insights gained from this exploratory work warrant further investigation in larger, mechanistic studies to define their precise roles and clinical utility.

## ORCID ID

Dongxue Zhang: 0000-0002-5714-7893

Teng Zhao: 0000-0002-1591-6770

Dehui Bi: 0009-0006-5708-2803

Jian Huang: 0009-0008-2059-4332

Pengxiang Zhao: 0000-0003-2432-6120

Bojun Wei: 0000-0003-2566-0139

## Funding

This research was funded by the Youth Fund of the National Natural Science Foundation of China (nº 82200872 Dongxue Zhang) and the Youth Foundation of Beijing Shijitan Hospital (nº 2018-q13, Dongxue Zhang).

## Conflicts of interest

The authors declare no conflicts of interest related to this study or its publication.

## Data availability statement

The authors declare that all data are available in repository.
